# No Association Between T-peak to T-end Interval on the Resting ECG and Long-Term Incidence of Ventricular Arrhythmias Triggering ICD Interventions

**DOI:** 10.3389/fphys.2020.01115

**Published:** 2020-08-31

**Authors:** Peter Michalek, Sasha Benjamin Hatahet, Martin Svetlosak, Peter Margitfalvi, Iveta Waczulikova, Sebastian Trnovec, Allan Böhm, Ondrej Benacka, Robert Hatala

**Affiliations:** ^1^Faculty of Medicine, Comenius University in Bratislava, Bratislava, Slovakia; ^2^Faculty of Medicine, Slovak Medical University in Bratislava, Bratislava, Slovakia; ^3^Department of Arrhythmias and Cardiac Pacing, The National Institute of Cardiovascular Diseases, Bratislava, Slovakia; ^4^Faculty of Mathematics, Physics and Informatics, Comenius University in Bratislava, Bratislava, Slovakia; ^5^Department of Acute Cardiology, The National Institute of Cardiovascular Diseases, Bratislava, Slovakia

**Keywords:** electrocardiography, primary prevention of sudden cardiac death, transmural dispersion of ventricular repolarization, T-peak to T-end, ventricular tachyarrhythmia

## Abstract

**Background and Objectives:**

Potential of using the T-peak to T-end (TpTe) interval as an electrocardiographic parameter reflecting the transmural dispersion of ventricular repolarization (TDR) to identify patients (pts.) with higher risk of malignant ventricular arrhythmias (MVA) for better selection of candidates for implantable cardioverter-defibrillator (ICD) in primary prevention (PP) of sudden cardiac death (SCD) remains controversial. The primary objective of this study was to investigate the relationship between the TpTe interval in patient’s preimplantation resting 12-lead electrocardiogram (ECG) and the incidence of MVA resulting in appropriate ICD intervention (AI). The secondary objective was to assess its relationship to overall mortality.

**Methods:**

A total of 243 consecutive pts. with severe left ventricular (LV) systolic dysfunction after myocardial infarction (MI) with a single-chamber ICD for PP of SCD from one implantation center were included. Excluded were all pts. with any other disease that could interfere with the indication of ICD implantation. Primarily investigated intervals were measured manually in accordance with accepted methodology. Data on ICD interventions were acquired from device interrogation during regular outpatient visits. Survival data were collected from the databases of health insurance and regulatory authorities.

**Results:**

We did not find a significant relationship between the duration of the TpTe interval and the incidence of MVA (71.5 ms in pts. with MVA vs. 70 ms in pts. without MVA; *p* = 0.408). Similar results were obtained for the corrected TpTe interval (TpTec) and the ratio of TpTe to QT interval (76.3 ms vs. 76.5 ms; *p* = 0.539 and 0.178 vs. 0.181; *p* = 0.547, respectively). There was also no significant difference between the duration of TpTe, TpTec and TpTe/QT ratio in pts. groups by overall mortality (71.5 ms in the deceased group vs. 70 ms in the survivors group; HR 1.01; 95% CI, 0.99–1.02; *p* = 0.715, 76.3 ms vs. 76.5 ms; HR 1.01; 95% CI, 0.99–1.02; *p* = 0.208 and 0.178 vs. 0.186; *p* = 0.116, respectively).

**Conclusion:**

This study suggests no significant association of overall or MVA-free survival with ECG parameters reflecting TDR (TpTe, TpTec) in patients with systolic dysfunction after MI and ICD implanted for primary prevention.

## Introduction

Under physiological conditions, the sequence of electrical activation and recovery of the heart is optimally synchronized in order to minimize heterogeneity of repolarization. The heterogeneity of repolarization processes (dispersion of repolarization) creates undesirable potential gradients between individual areas and layers of the myocardium, leading to a potentially proarrhythmogenic milieu.

In 1991, for the first time Sicouri and Antzelevitch described the electrophysiological properties of a hitherto unknown subpopulation of cardiomyocytes located in the deep subepicardial layer in both chambers of canine hearts (Sicouri and Antzelevitch, 1991). These M cells have distinct electrophysiological properties positioning them between cells of the cardiac conduction system and contractile cardiomyocytes. They are therefore a substrate for the existence of transmural dispersion of repolarization (TDR) across the ventricular myocardial wall. The end of the T wave on a resting 12-lead electrocardiogram (ECG) reflects the complete repolarization of the action potential (AP) of M cells, while its peak indicates the terminal phase of epicardial cells AP (Shimizu and Antzelevitch, 1998). Hence, the electrocardiographic parameter reflecting TDR is the interval from the peak of the T wave to its end (T-peak to T-end, TpTe). For the first time in the human heart, the presence of M cells was confirmed by Drouin et al. (1995), but the first clinical research was not conducted until 9 years later, when (Watanabe et al., 2004) demonstrated an association between TpTe prolongation (which reflects TDR accentuation) and a higher incidence of malignant ventricular arrhythmias. In the experiment, several authors described the electrophysiological mechanism of these processes (Antzelevitch et al., 1991; Yan et al., 2001; Gupta et al., 2008; Antzelevitch, 2010).

TpTe parameter subsequently became the subject of clinical research, but its results remain controversial (Lellouche et al., 2007; Letsas et al., 2009; Smetana et al., 2011; Morin et al., 2012; Porthan et al., 2013; Rosenthal et al., 2015). At present, even the very concept of association between TpTe and TDR is being questioned (Srinivasan et al., 2019), which only adds further doubts to its clinical validity (Malik et al., 2019).

Considering practical clinical application, it is most important to specify the potential of ECG measurements of the TpTe interval for the purpose of identifying patients at risk for malignant ventricular arrhythmias. Such a parameter would be desirable for improved patient selection for primary prevention of sudden cardiac death (SCD) using an implantable cardioverter-defibrillator (ICD). The unmet need for more precise patient selection for ICD implantation is not only due to the economic burden (García-Pérez et al., 2014), but also to the multifaceted ethical aspects (Strömberg et al., 2014) and well known complications of ICD therapy (Olde Nordkamp et al., 2016).

The primary objective of this study was to investigate the relationship between the duration of TpTe interval in patient’s resting 12-lead ECG and the incidence of malignant ventricular arrhythmias (MVA). A strictly defined population of patients with severe left ventricular systolic dysfunction after myocardial infarction (MI) with an ICD implanted for primary prevention of SCD and who did not require pacing was selected. In this population, the occurrence of malignant ventricular tachyarrhythmias can be very accurately identified by long-term permanent monitoring of heart rhythm by ICD. The secondary objective was to evaluate TpTe’s relationship to overall mortality and to analyze other clinical predictors of mortality.

## Materials and Methods

All consecutive patients who were implanted with an ICD in the years 2006–2015 in the primary prevention of SCD from the ICD register of the National Institute of Cardiovascular Diseases in Bratislava, Slovakia, were selected. Post-MI heart failure (HF) with left ventricular systolic dysfunction with an ejection fraction ≤ 35% was the principal inclusion criterion. To minimize the effect of other electrical disorders of the heart on the analyzed ECG parameters, only patients with single-chamber devices were included (as such disorders typically require implantation of dual-chamber or resynchronization ICD systems). Excluded from the analysis were patients:

• with ICD implanted for primary prevention of SCD due to another heart disease,
• with history of any arrhythmia and/or treatment with any antiarrhythmic drugs except beta blockers,
• with need of permanent ventricular pacing,
• with incomplete documentation and/or poor quality ECG recordings.

All patients were indicated and subsequently followed-up by the same team of physicians and the implantation procedure was in most cases performed by a single implanter (PM).

**FIGURE 1:**
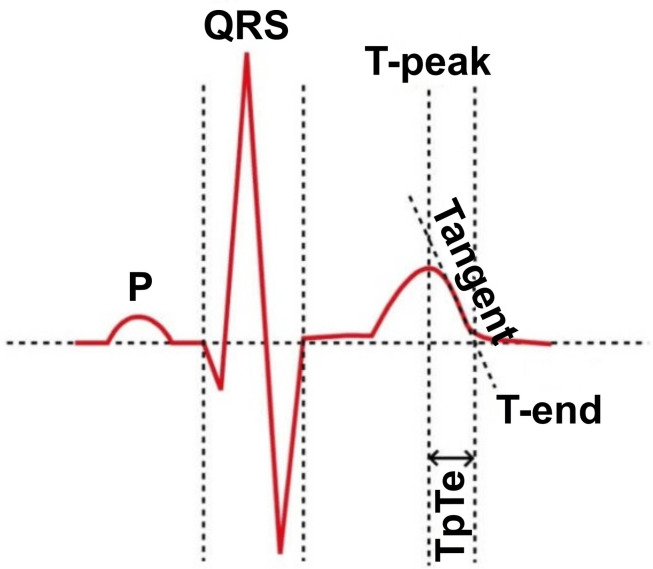
The Tangent method of TpTe measurement, adopted from Yılmaz (2017).

Last available ECG recording made prior to ICD implantation was used for measuring the ECG parameters. Standard 12-lead ECG recordings with a paper speed of 25 mm per second and an amplitude of 10 mm/mV were available and manual measurements of heart rate, QRS complex length, and QT interval were used. The QT interval was measured according to Goldenberg et al. (2006) and the duration of the QRS complex according to Turagam et al. (2013). The TpTe interval was measured from the top of the T wave to its end by the Tangent method (Rosenthal et al., 2017; Figure 1). The peak of the T wave was determined to be the most positive or the most negative amplitude from the isoelectric line. The intersection of the tangent with the descending part of the T wave and the isoelectric line was defined as the end of the T wave. All measurements were performed in lead V5 in accordance with the accepted methodology, leads V4 and V6 were used as alternative leads, respectively (Haarmark et al., 2009). The lead was omitted from the measurement if the most positive or negative deflection was less than 1.5 mm. The Bazett’s formula (Bazett, 1920) was used to calculate the corrected QT and TpTe intervals. All measurements were performed by single investigator (SBH). All recordings were scanned in high resolution on a Brother MFC-J5335DW scanner and measurements were performed in EP Calipers version 1.19 (EP Studios, Inc., Paris, FR).

Sustained ventricular tachycardia including torsades de pointes and ventricular fibrillation were considered as malignant ventricular arrhythmias. Data on ICD interventions (appropriate and inappropriate) were acquired from device interrogation during regular outpatient visits. All ICD therapies (antitachycardia pacing or shocks) for sustained ventricular tachycardia or ventricular fibrillation were considered as appropriate. Survival data were collected from the databases of health insurance and regulatory authorities.

### Statistical Analysis

Clinical and demographic data were analyzed using the methods of descriptive and inferential statistics. In the case of continuous data with a normal distribution tested by the Shapiro-Wilk test, we present the means and standard deviations (SD), the characteristics with an asymmetric distribution are described by the median and the first and third quartiles (IQR). Categorical data are presented as absolute and relative counts (a percentage of the respective total).

The between-group differences for normally distributed continuous variables were tested by a two-tailed unpaired Student’s t test. In the case of a significant deviation from the normal distribution a non-parametric Mann-Whitney test was used. Associations between categorical characteristics were tested by chi-square (χ^2^) tests. The effects of predictors on the primary and secondary endpoints are expressed as the respective odds ratios (OR), or hazard ratios (HR, as a form of immediate risk of the event). More precisely, OR (HR) expresses the ratio of a patient‘s odds of having the observed event (occurrence of malignant ventricular arrhythmias, or death), if the value of the analyzed predictor increases by 1, relative to his/her odds at the original value of the predictor. Consequently, if the OR (HR) > 1, the risk of the event among those in the comparison category (“cases”) will increase compared to those in the reference category (“controls”). The ORs (HRs) are presented together with the 95% confidence intervals (95% CI).

Statistical analyses were performed using Microsoft Office Excel 2010 (Microsoft Corporation, Redmond, US) and Stats Direct 3.2.7 (Stats Direct Ltd., Cheshire, United Kingdom). All *P*−values were considered significant at a two−tailed *P*−value of < 0.05.

## Results

From a total of 243 patients, appropriate ICD interventions were documented in 39.5% of patients, while 10.3% of patients had inappropriate interventions. The median time to first appropriate intervention (AI) was 23 months (IQR 7.5–43.0). The median follow-up was 62.6 months (IQR 41.4–91.6) and 69.5% of patients survived at the end of follow-up. Patients in the group with appropriate ICD interventions had a significantly better 3-year survival (92.7% vs. 82.3%, *P* = 0.022, Table 1).

**TABLE 1:** Clinical characteristics and follow-up data of patients grouped by the occurrence of appropriate ICD interventions.

Characteristics	Category (units)	Total	With AI ICD	Without AI ICD	*P*-value
Number of cases	(%)	243 (100%)	96 (39.5%)	147 (60.5%)	n.a.
Age	(Years)	58.8 ± 9.81	59.0 ± 9.39	58.3 ± 10.10	0.557
Gender	Female	30 (12.3%)	7 (7.3%)	23 (15.7%)	0.072
	Male	213 (87.7%)	89 (92.7%)	124 (84.3%)	
BMI	(kg/m^2^)	29.0 ± 4.79	29.5 ± 5.07	28.7 ± 4.60	0.184
Smoker	Yes	29 (11.9%)	11 (11.5%)	18 (12.2%)	0.853
	No	214 (88.1%)	85 (88.5%)	129 (87.8%)	
Diabetes mellitus	Yes	76 (31.3%)	27 (28.1%)	49 (33.3%)	0.479
	No	167 (68.7%)	69 (71.9%)	98 (66.7%)	
NYHA II and III	Yes	235 (96.7%)	94 (97.9%)	141 (95.9%)	0.485
	No	8 (3.3%)	2 (2.1%)	6 (4.1%)	
sBP	(mmHg)	126.1 ± 16.02	127.4 ± 15.48	125.3 ± 16.35	0.316
dBP	(mmHg)	79.1 ± 9.74	79.8 ± 9.51	78.6 ± 9.88	0.341
HR	(min^–1^)	68.0 (61.0–77.0)	67.5 (60.5–79.5)	68.0 (61.0–77.0)	0.773
LVEF	(%)	30 (25–32)	30 (25–31.5)	30 (25–32)	0.640
S-CREAT	(μmol/l)	92 (80–104)	94 (81–104)	91 (78–104)	0.643
CKD-EPI GFR	(ml/min/1.73 m^2^)	76.2 (64.6–92.5)	76.7 (65.7–91.3)	76.0 (62.9–93.2)	0.889

TpTe	(ms)	71.0 (62.0–83.0)	71.5 (62.0–80.0)	70.0 (62.0–85.0)	0.408
TpTec	(ms)	76.5 (65.6–89.8)	76.3 (65.5–85.4)	76.5 (65.7–91.2)	0.539
TpTe/QT	(ms/ms)	0.180 ± 0.038	0.178 ± 0.037	0.181 ± 0.040	0.547

Median follow-up	(months)	62.6 (41.4–91.6)	80.1 (54.3–99.6)	53.3 (35.1–77.5)	< 0.001
3-year survival rate	Dead	33 (13.6%)	7 (7.3%)	26 (17.7%)	0.022^a^
	Survived	210 (86.4%)	89 (92.7%)	121 (82.3%)	

*Continuous data are presented as means ± standard deviations, or as medians with lower – upper quartiles. Categorical data are presented as absolute counts with percent of column total. ^*a*^logrank test from Kaplan-Meier analysis. AI ICD, appropriate intervention(s) of ICD device; BMI, body mass index; NYHA, classification of heart failure symptoms according to New York Heart Association; sBP, systolic blood pressure; dBP, diastolic blood pressure; HR, heart rate; LVEF, left ventricular ejection fraction; S-CREAT, serum creatinine; CKD-EPI GFR, estimated glomerular filtration rate according to Chronic Kidney Disease Epidemiology Collaboration; TpTe, T-peak to T-end interval; TpTec, corrected T-peak to T-end interval; TpTe/QT, T-peak to T-end and QT intervals ratio; n.a., not applicable.*

**FIGURE 2:**
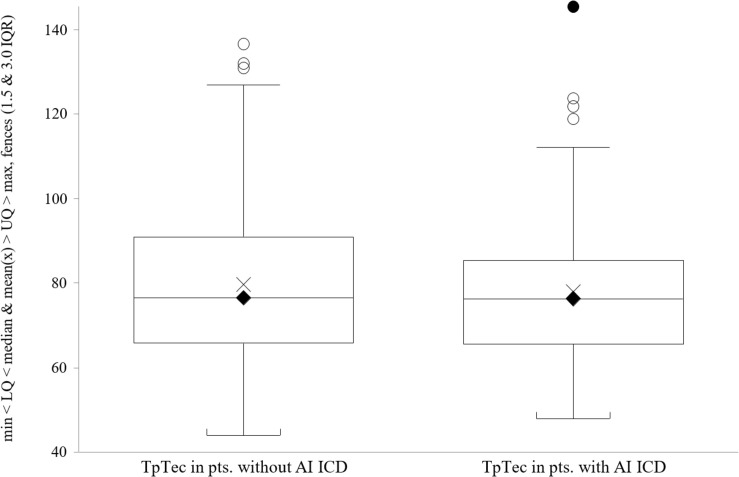
TpTec interval in patients without/with appropriate ICD interventions.

We did not find a significant relationship between the duration of the TpTe interval and the incidence of appropriate ICD interventions when analyzing TpTe differences in patient groups by primary endpoint (71.5 ms, IQR 62.0–80.0 in the group with AI vs. 70.0 ms, IQR 62.0–85.0 in the group without AI, P = 0.408). Similar results were obtained for the corrected TpTe interval (TpTec) and the ratio of TpTe to QT interval (76.3 ms, IQR 65.5–85.4 in the group with AI vs. 76.5 ms, IQR 65.7–91.2 in group without AI, *P* = 0.539 and 0.178 ± 0.037 vs. 0.181 ± 0.040, *P* = 0.547, respectively, Table 1). The distribution of TpTec data in both groups is presented in Figure 2.

Results of fitting the multinomial logistic regression models to the data did not reveal any significant explanatory variables for the event of appropriate intervention, but gender. We have found male gender to be significantly associated with higher proportion of appropriate ICD interventions on the univariate analysis (HR = 2.29; 95% CI, 1.06–4.94; *P* = 0.034) and this contribution was maintained also on the multivariate analysis (HR = 2.32; 95% CI, 1.06–5.09; *P* = 0.035).

There was also no significant difference between the duration of TpTe, TpTec and TpTe/QT ratio in patient groups by overall mortality (71.5 ms, IQR 62.0–80.0 in the deceased group vs. 70.0 ms, IQR 62.0–85.0 in the survivors group, HR 1.01; 95% CI, 0.99–1.02; *P* = 0.715 and 76.3 ms, IQR 65.5–85.4 vs. 76.5 ms, IQR 65.7–91.2, HR 1.01; 95% CI, 0.99–1.02; *P* = 0.208 and 0.178 ± 0.038 vs. 0.186 ± 0.039; *P* = 0.116 respectively, Table 2). From other ECG indices, the only significant difference was observed between the duration of uncorrected QT intervals in patient groups by overall mortality (396.4 ± 43.08 ms in the deceased group vs. 415.0 ± 46.04 ms in the survivors group, *P* = 0.003, Table 3).

**TABLE 2:** Clinical characteristics and follow-up data of patients grouped by overall survival.

Characteristics	Category (units)	Dead	Survived	HR (95% CI)	*P*-value
Number of cases	(%)	74 (30.5%)	169 (69.5%)	n.a.	n.a.
Age	(Years)	62.7 ± 8.46	56.7 ± 9.82	1.06 (1.04–1.09)	< 0.001
Gender	Female	12 (16.2%)	18 (10.7%)	1.31 (0.70–2.42)^a^	0.399
	Male	62 (83.8%)	151 (89.3%)		
BMI	(kg/m^2^)	27.9 ± 5.17	29.5 ± 4.53	0.94 (0.89–0.98)	0.009
Smoker	Yes	8 (10.8%)	21 (12.4%)	0.84 (0.40–1.75)^a^	0.642
	No	66 (89.2%)	148 (87.6%)		
Diabetes mellitus	Yes	30 (40.5%)	46 (27.2%)	1.67 (1.05–2.66)^a^	0.031
	No	44 (59.5%)	123 (72.8%)		
NYHA II and III	Yes	74 (100%)	161 (95.3%)	∞	0.110
	No	0 (0%)	8 (4.7%)		
sBP	(mmHg)	125.5 ± 14.77	126.4 ± 16.57	0.99 (0.98–1.01)	0.229
dBP	(mmHg)	78.3 ± 7.95	79.4 ± 10.43	0.99 (0.96–1.01)	0.194
HR	(min^–1^)	70 (64–86)	67 (60–75)	1.02 (1.00–1.03)	0.024
LVEF	(%)	27.7 ± 4.86	29.8 ± 5.44	0.93 (0.89–0.97)	0.001
S-CREAT	(μmol/l)	96 (81–113)	91 (79–102)	1.01 (1.01–1.02)	< 0.001
CKD-EPI GFR	(ml/min/1.73 m^2^)	71.9 (54.3–88.0)	77.7 (67.8–94.5)	0.98 (0.97–0.99)	< 0.001

TpTe	(ms)	71.5 (62.0–80.0)	70 (62.0–85.0)	1.01 (0.99–1.02)	0.715
TpTec	(ms)	76.3 (65.5–85.4)	76.5 (65.7–91.2)	1.01 (0.99–1.02)	0.208
TpTe/QT	(ms/ms)	0.178 ± 0.038	0.186 ± 0.039	n.a.	0.116

Median follow-up	(Months)	38.2 (27.2–63.0)	74.7 (52.1–96.3)	n.a.	< 0.001
Appropriate intervention of ICD device (at least 1)^b^	Yes	28 (37.8%)	68 (40.2%)	0.68 (0.42–1.09)^a^	0.108
	No	46 (62.2%)	101 (59.8%)		
Median time to first appropriate intervention	(Months)	14.6 (5.0–23.0)	27.8 (7.8–48.0)	n.a.	0.007

*Continuous data are presented as means ± standard deviations, or as medians with lower – upper quartiles. Categorical data are presented as absolute counts with percent of column total. ^*a*^The ratio of the odds of having an event in the upper row category to the odds of having an event in the lower row category of the predictor variable. ^*b*^At the end of the follow-up. HR, hazard ratio; BMI, body mass index; NYHA, classification of heart failure symptoms according to New York Heart Association; sBP, systolic blood pressure; dBP, diastolic blood pressure; HR, heart rate; LVEF, left ventricular ejection fraction; S-CREAT, serum creatinine; CKD-EPI GFR, estimated glomerular filtration rate according to Chronic Kidney Disease Epidemiology Collaboration; TpTe, T-peak to T-end interval; TpTec, corrected T-peak to T-end interval; TpTe/QT, T-peak to T-end and QT intervals ratio; AI ICD, appropriate intervention(s) of ICD device; n.a., not applicable.*

**TABLE 3:** Duration of QRS complex and QT(c) intervals in studied patients groups.

Characteristics	Units	Total	With AI ICD	Without AI ICD	*P*-value
Number of cases	(%)	243 (100%)	96 (39.5%)	147 (60.5%)	n.a.
QRS	(ms)	111.7 ± 20.00	113.2 ± 20.48	110.7 ± 19.25	0.328
QT	(ms)	409.3 ± 45.88	404.9 ± 40.80	412.2 ± 48.83	0.228
QTc	(ms)	438.8 ± 35.93	439.4 ± 38.96	437.9 ± 33.93	0.747

**Characteristics**	**Units**	**Total**	**Dead**	**Survived**	***P*-value**

Number of cases	(%)	243 (100%)	74 (30.5%)	169 (69.5%)	n.a.
QRS	(ms)	111.7 ± 20.00	114.7 ± 20.95	110.3 ± 19.48	0.116
QT	(ms)	409.3 ± 45.88	396.4 ± 43.08	415.0 ± 46.04	0.003
QTc	(ms)	438.8 ± 35.93	434.5 ± 35.69	440.7 ± 35.97	0.215

*Continuous data are presented as means ± standard deviations. Categorical data are presented as absolute counts with percent of column total. AI ICD, appropriate intervention(s) of ICD device; n.a., not applicable.*

Significant factors affecting mortality included: higher age (HR 1.06; 95% CI, 1.04–1.09; *P* < 0.001), lower ejection fraction of left ventricle (HR 0.93; 95% CI, 0.89–0.97; *P* = 0.001), higher serum creatinine (HR 1.01; 95% CI, 1.01–1.02; *P* < 0.001), lower glomerular filtration rate (HR 0.98; 95% CI, 0.97–0.99; *P* < 0.001), higher heart rate (HR 1.02; 95% CI, 1.00–1.03; *P* = 0.024) and lower BMI (HR 0.94; 95% CI, 0.89–0.98; *P* = 0.009). There was a 1.7-fold higher risk of death in patients with diabetes (HR 1.67; 95% CI, 1.05–2.66; *P* = 0.031). No significant difference in survival between smokers and non-smokers was observed (HR 0.84; 95% CI, 0.40–1.75; *P* = 0.642, Table 2).

## Discussion

According to our knowledge, this work is the first attempt to analyze the relationship of the TpTe parameter and the incidence of ventricular tachyarrhythmias in a strictly defined population of patients after myocardial infarction with a single-chamber ICD without the need for permanent ventricular pacing. In our analysis, significant differences in the monitored ECG parameters (TpTe, TpTec) as well as in the ratio of TpTe to QT intervals between the patients with and without malignant ventricular arrhythmias were not observed. The occurrence of malignant ventricular arrhythmias was assessed based on the presence of appropriate ICD interventions in the event of such arrhythmias. Differences in ECG parameters between the groups of surviving and deceased patients were also not demonstrated.

The results of this analysis in an exactly defined and monitored patient population did not confirm an association between prolongation of TpTe (which should reflect increased TDR) and a higher incidence of malignant ventricular arrhythmias. Thus, our work ranks among those who failed to prove any predictive value of the TpTe interval for overall mortality or for better selection of patients with a higher risk for malignant arrhythmias (Smetana et al., 2011; Porthan et al., 2013). In our work we have analyzed only post-MI patients with chronic HF without the need for permanent cardiac pacing or resynchronization therapy. Exclusion of patients with other proarrhythmogenic comorbidities is another potential advantage of our cohort. Its homogeneity is also reflected in the consistency of the severity of HF quantitatively expressed by the degree of systolic dysfunction of the left ventricle. Thus, in the light of our results, left ventricle ejection fraction remains the only clinical quantitative parameter used for selection of candidates for the primary prevention of SCD.

The long-term follow-up of patients in this dataset provides some interesting clinical observations: Only less than 12% of patients were smokers, quite in contrast with studies with higher proportion of smokers (Lellouche et al., 2007; Porthan et al., 2013). We anticipate a strong psychological impact of the circumstances arising from the diagnostic and treatment process in patients with coronary heart disease and HF, which may have resulted in smoking cessation. However, it is also possible that patients concealed smoking out of an (unfounded) fear of refusing to provide costly treatment. Smoking in our cohort did not affect overall mortality. Such a “smoker’s paradox” is a well-known phenomenon in the population of patients with HF (Fonarow et al., 2008). However, recent analyses consider this paradox to be the result of a statistical bias in the impact of smoking in a population with many risk factors (Doi et al., 2019). Similarly, our results (difference in BMI in groups by overall mortality + 1.64 kg/m^2^ in the group of surviving patients, 95% CI for the difference between means 0.34–2.94) agree with the known “obesity paradox” (Padwal et al., 2013). Systolic and diastolic blood pressure values indicate a good control of arterial hypertension in the monitored population, which however, may be caused by the phenomenon of “decapitated hypertension” in HF patients. The higher risk of death in patients with diabetes is consistent with the trend of their higher mortality in large population-based studies (Raghavan et al., 2019). It is clinically very interesting to find that lower glomerular filtration rate (GFR) is already a significant predictor of overall mortality with minimal renal impairment (KDIGO category G2 with normal or only minimally elevated serum creatinine). Considering large meta-analyses (van der Velde et al., 2011), both overall and cardiovascular mortality increased in proportion to renal impairment starting with GFR of 60 ml/min and lower. The median GFR in our group of deceased patients was 71.9 ml/min (IQR 54.3–88.0), which indicates a very significant effect of renal function on the prognosis of patients in our population.

The significant association of male gender with higher proportion of appropriate ICD interventions is nowadays a well-known phenomenon (Sticherling et al., 2018; Santangeli et al., 2010), and in the context with similar gender mortality suggest a smaller impact of sudden cardiac death on overall survival in women in this population. Greenlee et al. (2018) analyzed ICD treatment in patients from larger registries (a total of 2,540 ICD recipients) in the primary prevention of SCD and found that more than two-thirds of patients never received ICD-mediated intervention while more than three quarters of patients never received an appropriate therapy (ICD intervention triggered by ventricular tachycardia or ventricular fibrillation). However, the median follow-up in the mentioned work was less than two and a half years, while in our work more than 5 years. Almost 40% of patients received appropriate treatment in our cohort, and the median time to the first appropriate intervention was less than 2 years (23 months). Such a number of patients with appropriate ICD intervention indicates a very precise selection of candidates for primary preventive implantation of ICD at participating implantation center. Consistent with this finding is the fact that approximately 10% of our patients had inappropriate interventions, which is significantly less compared to the latter work (12% at half follow-up), probably mainly due to explicit elimination of patients with atrial fibrillation. This is in line with several clinical studies that have not confirmed the original intuitive hypothesis that the use of dual-chamber ICDs will reduce inappropriate interventions as well as overall mortality, as systems with added atrial electrode can better differentiate ventricular from supraventricular tachyarrhythmias. The preference for dual-chamber ICDs did not lead to a reduction in the rate of inappropriate treatment, and also was associated with a higher risk of peri- and post procedural complications and the need for earlier replacement of the generator (Theuns et al., 2008; Lee et al., 2010; Peterson et al., 2013; Defaye et al., 2017).

### Limitations of the Study

As there is no uniform methodology for measuring the TpTe interval, procedure of any method described so far will always limit the investigation of the subject interval in relation to clinical outcomes. Because the study intervals were measured for each patient only at the beginning of the follow-up (changes in intervals over time are not captured), it is not statistically possible to describe direct relationships between the observed characteristics and outcomes, but “only” differences in the characteristics in the selected groups of patients.

The evaluation of an appropriate ICD intervention as a surrogate of the SCD is very often discussed. This is related to the knowledge acquired from the monitoring data from the ICD, which very convincingly prove that even malignant ventricular tachyarrhythmia (including ventricular fibrillation) can terminate spontaneously. This is in contrast to the “traditional” perception of these arrhythmias as “irreversible” and lethally ending without therapeutic intervention. Therefore, the extrapolation of appropriate ICD interventions as an exact measure of the incidence of SCD cannot be fully accepted, but it is a clinically meaningful approximation.

Other relative limitations include the retrospective monocentric design of the study, as well as the absence of randomization, assessment of infarct location, and separate monitoring of arrhythmic mortality.

## Conclusion

The results of our retrospective study with long-term follow-up of post-MI patients with heart failure undergoing ICD implantation for primary prevention do not suggest any significant association of electrocardiographic parameters reflecting transmural dispersion of ventricular repolarization (TpTe, TpTec) with overall and arrhythmia-free survival. Differences in some known clinical prognostic factors associated with overall survival in this population were found.

## Data Availability Statement

The data analyzed in this study is subject to the following licenses/restrictions: The dataset is copyright protected by ownership of The National Institute of Cardiovascular Diseases, Bratislava, Slovakia. Requests to access these datasets should be directed to RH, robert.hatala@nusch.sk.

## Ethics Statement

The studies involving human participants were reviewed and approved by the Etická komisia NÚSCH, a.s., Bratislava. The patients/participants provided their written informed consent to participate in this study.

## Author Contributions

PMi contributed to the design, methodology, dataset, statistical analyses, and interpretation of the manuscript. SH contributed to the dataset and the intervals measurements. MS contributed to the design, manuscript, and the revision. PMa contributed to the methodology and devices implantation. IW contributed to the statistical analyses and interpretation. ST contributed to the dataset. AB contributed to the design and methodology. OB contributed to the dataset. RH contributed to the design, methodology, manuscript, and revision. All authors contributed to the article and approved the submitted version.

## Conflict of Interest

The authors declare that the research was conducted in the absence of any commercial or financial relationships that could be construed as a potential conflict of interest. The handling editor declared a past co-authorship with two of the authors MS and RH.
